# Silencing of STIM1 attenuates hypoxia-induced PASMCs proliferation via inhibition of the SOC/Ca^2+^/NFAT pathway

**DOI:** 10.1186/1465-9921-14-2

**Published:** 2013-01-05

**Authors:** Xianhua Hou, Jian Chen, Yongjun Luo, Fuyu Liu, Gang Xu, Yuqi Gao

**Affiliations:** 1 Department of Pathophysiology and high altitude physiology, College of high altitude military medicine, Third Military Medical University, Chongqing, China; 2 Key Laboratory of High Altitude Medicine, Third Military Medical University, Ministry of Education, 400038, Chongqing, China; 3 Key Laboratory of High Altitude Medicine of PLA, 400038, Chongqing, China

**Keywords:** Stromal interaction molecule 1, RNA interference, Pulmonary hypertension, Hypoxia, Cell proliferation

## Abstract

**Background:**

Stromal interaction molecule 1 (STIM1) is a newly discovered Ca^2+^ sensor on the endoplasmic reticulum which is an indispensable part in the activation of store-operated Ca^2+^ channels (SOC). Recent studies demonstrate that SOC of pulmonary smooth muscle cells (PASMCs) were upregulated by chronic hypoxia which contribute to the enhanced pulmonary vasoconstriction and vascular remodeling. However, the exact role of STIM1 in the development of chronic hypoxic pulmonary hypertension(HPH) remains unclear.

**Methods:**

In this study we investigated the cellular distribution and expression of STIM1 by immunofluorescence, qRTPCR and Western blotting methods in Wistar rat distal intrapulmonary arteries under normal and chronic hypobaric hypoxic conditions. In vitro, Wistar rat PASMCs were isolated and cultured. PASMCs were transfected with siRNA targeting STIM1 gene by liposome. The expression of STIM1 protein was detected by Western blotting. [^3^H]-thymidine ([^3^H]-TdR) incorporation were performed to detect PASMCs proliferation. The cell cycle was analyzed by flow cytometry. The SOC-mediated Ca^2+^ influx was calculated by Ca^2+^ fluorescence imaging and the nuclear translocation of NFATc3 was determined by immunofluorescence and Western blot analysis of nuclear extracts.

**Results:**

We found that during the development of HPH and the initiation of vascular remodeling, the mRNA and protein expression levels of STIM1 significantly increased in the distal intrapulmonary arteries. Moderate hypoxia significantly promotes PASMCs proliferation and cell cycle progression. Silencing of STIM1 significantly decreased cellular proliferation and delayed the cell cycle progression induced by hypoxia. Silencing of STIM1 also significantly decreased SOC-mediated Ca^2+^ influx and inhibited the nuclear translocation of NFATc3 in hypoxic PASMCs.

**Conclusion:**

Our findings suggest that chronic hypobaric hypoxia upregulates the expression of STIM1 in the distal intrapulmonary arteries which plays an important role in the hypoxia-induced PASMCs proliferation via SOC/Ca^2+^/NFAT pathway and may represent a novel therapeutic target for the prevention of hypoxia pulmonary hypertension.

## Background

Chronic exposure to hypobaric hypoxia cause hypoxic pulmonary hypertension (HPH), which is characterized by pulmonary vasoconstriction (HPV) and vascular remodeling [1]. The relationship between Ca^2+^ and HPH has been throughly investigated. Hypoxia inhibits the Kv channels, leading to cell membrane depolarization, and triggers Ca^2+^ influx from the L-type Ca^2+^channels. The increased Ca^2+^ levels cause pulmonary arterial smooth muscle cells (PASMCs) to constrict and proliferate which contributes to the development of pulmonary hypertension [2-5]. However, recent studies suggested that the store-operated Ca^2+^ channel (SOC) in addition to L-type Ca^2+^ channels are also involved in chronic hypoxic pulmonary hypertension [6]. The activation of SOC is triggered by a reduction in the concentration of SR Ca^2+^, which can be depleted by inhibiting sarcoendoplasmic reticulum Ca^2+^ ATPases with thapsigargin (TG) [7,8]. Recently, an RNAi-based screening approach revealed that a novel membrane-spanning protein, stromal interaction molecule 1 (STIM1), was required for the activation of SOC [9,10]. STIM1 is a 90-kDa type-I transmembrane Ca^2+^-binding protein with a luminal helix-turn-helix EF-hand Ca^2+^-sensing module followed by a sterile α motif (SAM) in the intraluminal NH_2_-terminal region. Decreased binding of Ca^2+^ to the EF hand has been shown to lead to the oligomerization of STIM1 followed by translocation of the multimers to membrane-adjacent ER areas where STIM1 can activate Ca^2+^ influx [9,11-13]. It has been reported that STIM1 expressed in the pulmonary arteries and played important roles in the activation of SOC in rat PASMCs [14]. However, The functions of STIM1 involved in HPH are still obscure. In this study, we present in vivo and in vitro evidence showing that hypobaric upregulates the expression of STIM1 in rat distal intrapulmonary arteries which plays an important role in the hypoxia-induced PASMCs proliferation via SOC/Ca^2+^/NFAT pathway and may represent a novel therapeutic target for the prevention of hypoxia pulmonary hypertension.

## Materials and methods

### Animals

All of the protocols and surgical procedures were approved by the Institutional Animal Use Committee of the Third Military Medical University and were in accordance with the National Institutes of Health and the American Physiological Society guidelines. Adult male Wistar rats (6 – 7 weeks old, 220 – 250 g) were placed for 21 days in a chamber that was depressurised to 380 mmHg with a 12-hour light–dark cycle. Age-matched controls were maintained in normal room air. Each group consisted of 15 experimental animals. The methods that were used to isolate the rat lungs were similar to those previously reported [15]. In brief, prior to lung isolation, mean pulmonary arterial pressures were measured as previously described [16]. After euthanizing the rats, the thorax was immediately opened and the heart and lungs were removed. The hearts were dissected to remove the right ventricle (RV) free wall and the left ventricle plus septum (LV + S), and the weight ratio of RV/(LV + S) was used as an index of RV hypertrophy. The distal intrapulmonary arteries were dissected from the lungs and frozen in liquid nitrogen for subsequent examination.

### Morphological preparation and examination

The lungs was perfused with 4% paraformaldehyde (PFA), inflated by infusion of 4% PFA at a constant pressure of 25 cm H_2_O through the cannula inserted in the trachea, fixed in 4% PFA overnight at 4°C and then embedded in OCT, and subsequently cut into 10-μm-thick sections for hematoxylin and eosin staining. Following H&E staining, Images of individual pulmonary arteries were captured using a digital camera, mounted on a light microscope, and linked to a computer. the ratio of vessel wall area to total area (WA%) and the ratio of pulmonary arteriole wall thickness to vascular external diameter (WT%) were measured using the Image-Pro Plus 5.1 software.

### Quantitative real-time polymerase chain reaction (qRTPCR)

Total RNA from the intrapulmonary arteries (isolated as mentioned above) was isolated using the RNA simple Total RNA Kit (Tiangen), according to the manufacturer’s protocol. Then, the total RNA was reverse-transcribed to cDNA using the PrimeScript® RT reagent kit (TAKARA). Real-time PCR was performed using the SYBR® Premix Ex Taq™ II kit (TAKARA). The primer sequences for STIM1 were 5^′^-CGTCCGCAACATCCACAAG-3^′^ (forward) and 5^′^-CCATAGGTCCTCCACGCT-3^′^ (reverse). The primer sequences for β-actin were 5^′^-ACGGTCAGGTCATCACTATC-3^′^ (forward) and 5^′^-TGCCACAGGATTCCATACC-3^′^ (reverse). The amplification conditions consisted of 1 cycle at 95°C for 30 s and 40 cycles of 95°C for 5 s, 60°C for 20 s, and 72°C for 15 s. Melting curve analyses were performed at conditions of 95°C for 1 min and then 55°C for 1 min, which were followed by 80 increments of +0.2°C at 10-s intervals. The relative concentrations of each transcript were calculated using the standard curve method.

### Double-immunofluorescence staining

The isolated lungs were formaldehyde fixed (4% in PBS), cryoprotected with 30% sucrose in PBS, embedded in OCT media, and then frozen. Cryostat sections (10 μm) were permeabilised and blocked for nonspecific binding and then incubated in 0.2% gelatin in PBS with the following primary antibodies at 4°C overnight: mouse monoclonal anti-STIM1 (1:100) (BD Bioscience) and rabbit polyclonal anti-α-smooth muscle actin (1:250) (ABcam). The secondary antibodies [anti-mouse Cy3 and anti-rabbit FITC (1:500) (Biyuntian, China)] were prepared in 0.2% gelatin in PBS and were applied to the sections for 1 h at 37°C. Nuclei were stained using DAPI (1:1,000 in PBS; Biyuntian, China). Images were taken using a confocal laser scanning microscope (Leica TCS SP5, Germany).

### Isolation and culture of PASMCs

For isolation of the PASMCs, the adventitia of freshly excised distal (>4th generation) intrapulmonary arteries from adult male Wistar rats were removed. Vascular segments were then cut open, and the endothelium was removed by gently scraping the luminal surface of the vessel. Rat PASMCs were cultured as previously described [17]. In brief, the arteries were allowed to recover for 30 min in cold (4°C) physiological salt solution (PSS) that contained 130 mM NaCl, 5 mM KCl, 1.2 mM MgCl_2_, 10 mM HEPES, and 10 mM glucose. This was followed by 20 min in reduced-Ca^2+^ PSS (20 μM CaCl_2_) at room temperature. The tissue was then digested at 37°C for 20 min in reduced-Ca^2+^ PSS containing collagenase (type I, 1,750 U/ml), papain (9.5 U/ml), bovine serum albumin (2 mg/ml), and dithiothreitol (1 mM). Cells were grown in DMEM supplemented with 10% FBS and were passaged at a 1:3 ratio with trypsin treatment. The purity of the PASMCs in the primary cultures was confirmed using the specific mAb against smooth muscle α-actin. Cells that had been passaged 3 to 8 times and were at 80% confluence were used for all experiments.

### RNA interference

Small interfering RNA (siRNA) targeting STIM1 (siSTIM1) were designed as previously described [18] and synthesised by Genepharma. Scrambled (nonsense) siRNA (Genepharma) was used as negative control siRNA. Cells were transfected with siSTIM1 or Scrambled siRNA (final concentration of siRNA was 90 nM) (Invitrogen, Carlsbad, CA) using Lipofectamine RNAiMAX (Invitrogen) according to the manufacturer’s instructions. After 24 h, Cell growth was arrested by replacing medium with serum-free DMEM for 24 h under normoxic conditions. Growth-arrested cells were further incubated under either normoxic(21% O_2_) or hypoxic conditions (3% O_2_) for 24 h.

### Western blotting

Forty-eight hours after transfection, cells were collected for protein isolation. The cultured cells were washed twice with ice-cold PBS and lysed on ice in RIPA lysis buffer containing freshly added protease and phosphatase inhibitor cocktails. After 15 min of incubation, the cell lysate was collected by centrifuging the cells for 5 min(16,000 × g) at 4°C. For nuclear and cytoplasmic fractions, adherent cells were washed in PBS, and the cytoplasmfraction was prepared by the addition of buffer C (10 mM Tris, pH 7.6, 10 mM KCl, 1.5 mM MgCl_2_, 1% (v/v) Triton X-100, 1 mM dithiothreitol, 0.2 mM Na_3_VO_4_, 0.4 mM phenyl-methylsulfonyl fluoride, 10 μg/ml leupeptin, and 0.2 mM NaF). After a 15-min incubation on ice, lysates were spun for 5 min (10,000 × g) at 4°C. Supernatant containing the cytoplasm fraction was saved, and the pellet, containing the nuclear fraction, was washed once with buffer C and resuspended in buffer N (20 mM Tris, pH 7.6, 160 mM KCl, 1.5 mM MgCl_2_, 10% (v/v) glycerol, 1 mM dithiothreitol, 0.2 mM Na_3_VO_4_, 0.4 mM phenylmethylsulfonyl fluoride, 10 μg/ml leupeptin, and 0.2 mM NaF). Nuclear lysates were incubated for 30 min on a rotating platformat 4°C and spun (16,000 × g) for 15 min at 4°C. The amount of total protein was determined using a BCA protein assay kit (Pierce, Rockford, IL, USA). An equal amount of total protein (30-50 μg) was loaded and separated by SDS-PAGE. The protein was transferred to polyvinylidene difluoride membranes and was then blocked and probed with the appropriate antibodies. Monoclonal Abs against STIM1 (BD Bioscience), Lamin B1 (Santa Cruz) and β-actin (Santa Cruz) or a polyclonal antibody against NFATc3 (Santa Cruz) were used as primary Abs. The membranes were then washed for 15 min 3 times and incubated with horseradish peroxidase-conjugated goat anti-rabbit or anti-mouse IgG for 1 h. Bound antibodies were detected using an enhanced chemiluminescence system following the manufacturer’s instructions. Densitometric signals were quantified by Quantity One software.

### Determination of cell proliferation

PASMC proliferation was quantified by [3H]-thymidine incorporation, as described previously [19]. Briefly, 1 μCi/well [3H]-thymidine was added for the final 6 hours of cell culture, after which the cells were removed from the wells with trypsin digestion. The incorporated [3H]-thymidine was precipitated with 10% trichloroacetic acid and counted using a liquid scintillation counter. The experiments were repeated three times and results from five wells per experiment were determined and expressed as the average of the counts.

### Cell cycle and DNA analyses

PASMCs were harvested by trypsin-EDTA treatment and were fixed in 70% ethanol. The ethanol was removed, and the cells were incubated in PBS containing RNase at 37°C for 30 min. Next, the cells were stained with propidium iodide (50 μg/ml) and suspended in PBS for 30 min on ice. DNA fluorescence was measured by flow cytometry using an EPICS XL cytometer (Beckman Coulter, CA).

### Measurement of SOCE

The [Ca^2+^]i in PASMCs was measured using the Ca^2+^-sensitive fluorescent indicator fluo-4/AM. Cells were loaded with fluo-4 at 37°C for 30 min using a Ca^2+^-free physiological salt solution (D-HANKS) containing 5 μM fluo-4/AM. The fluo-4-loaded cells were then perfused with a Ca^2+^-free physiological salt solution containing the following components: 0.5 mM EGTA (Sigma Chemical, St Louis, MO)to chelate residual Ca^2+^, 5 μM nifedipine (Sigma Chemical, St Louis, MO) to prevent calcium entry through L-type voltage-operated Ca^2+^ channels (VOCC), and 10 μM cyclopiazonic acid (CPA; Sigma Chemical) to deplete SR Ca^2+^ stores. The [Ca^2+^]i was determined before and after the restoration of extracellular [Ca^2+^] to 2.5 mM. SOCE was evaluated by measuring the peak increase in [Ca^2+^]i caused by the restoration of extracellular Ca^2+^ in the continued presence of nifedipine and CPA.

### Immunofluorescence microscopy

Cells were fixed for 30 min at room temperature with 4% formaldehyde in Dulbecco's phosphate-buffered saline (DPBS). Next, the cells were incubated with 0.2% Triton X-100 in DPBS for 15 min at room temperature. The cells were blocked with blocking solution (10% goat serum in DPBS) for 1 h and then incubated with the primary antibodies (NFATc3, sc-8321, Santa Cruz Biotechnology, Santa Cruz, CA, USA) for 1 h at room temperature followed by the fluorescent-conjugated secondary antibody (FITC-conjugated AffiniPure goat anti-rabbit IgG, Beijing Zhongshan Golden Bridge Biological Technology Company, Beijing, China) for 90 min at room temperature. Nuclear staining was performed using DAPI (Biyuntian, China). The fluorescence was examined using a Leica laser scanning confocal microscope (Leica TCS SP5, Germany).

### Statistical methods

Numerical data were expressed as the means ± SEM. The SPSS10.0 software was used for the statistical analysis. An ANOVA was used with Scheffe’s multiple comparison tests for multiple groups and student’s *t*-test was used for two groups. P values < 0.05 were regarded as statistically significant.

## Results

### Morphological characteristics and alterations

**Figure 1:**
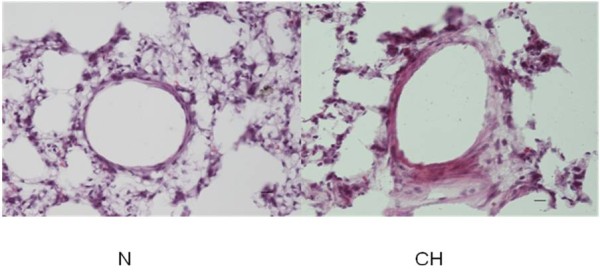
**H&E-stained sections of small pulmonary arteries from the lungs of control rats (N) and rats exposed to hypobaric hypoxia for 21 days (CH).** The results show representative sections from at least three rats per group for each time point. Scale bar = 10 μm.

The mean pulmonary arterial pressures were 18.5 ± 2.3 mmHg and 31.5 ± 3.7 mmHg for the controls and the animals exposed to hypoxic conditions for 21 days, respectively (p < 0.05, n = 10). Furthermore, the ventricular weight measurements revealed the RV/(LV + S) ratios to be 0.26 ± 0.02 for control rats and 0.42 ± 0.03 for rats exposed to hypoxia for 21 days (p < 0.05, n = 10). The histological H&E staining demonstrated that the ratio of pulmonary arteriole wall thickness to vascular external diameter (WT%) were 16.2 ± 5.2 and 34.8 ± 7.4 for the control and rats exposed to hypoxia for 21 days respectively (p < 0.05, n = 3). The ratio of vessel wall area to total area (WA%) were 29.7 ± 7.9 and 56.2 ± 8.8 for the control and rats exposed to hypoxia for 21 days respectively (p < 0.05, n = 3). These data indicated that the wall area and wall thickness percentages were significantly increased in the hypoxia-treated group compared to the control group (Figure 1).

### Effects of hypoxia on the expression of STIM1 in rat distal intrapulmonary arteries

**Figure 2:**
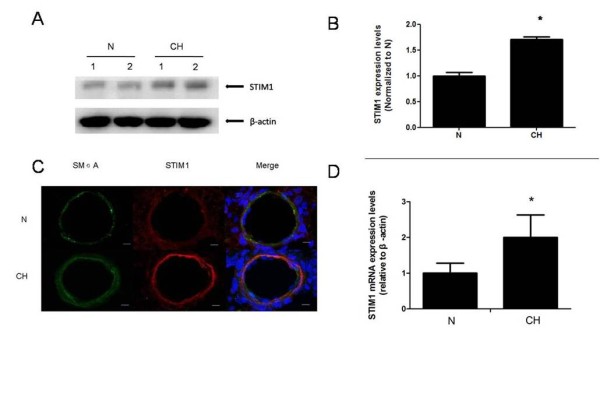
**The effects of chronic hypoxia on STIM1 mRNA and protein expression.** Representative Western blot images (**A**) and summarized data (**B**) for STIM1 proteins in distal (>4th generation) intrapulmonary arteries from control rats (N) and rats exposed to hypobaric hypoxia for 21 days (CH). β-actin was used as a loading control. Graph shows protein expression of STIM1 normalized to an average level in N. The results are expressed as the mean ± SEM for five experiments *: *P* < 0.05 compared to the N group. (**C**) STIM1 (red) and α-smooth muscle actin (ACTA2) (green) immunofluorescence staining in small pulmonary arteries from N and CH. Nuclei are counterstained with DAPI (blue). Scale bar = 10 μm. The results shown are from a single experiment and are representative of three separate experiments. (**D**) Analysis of STIM1 mRNA expression in distal (>4th generation) intrapulmonary arteries from N and CH. Parallel amplification of the rat housekeeping β-actin gene was used as an internal control. The results are expressed as the mean ± SEM for five experiments. *: *P* < 0.05 compared to the N group.

To detect the effects that hypoxia had on the expression of STIM1 in rat distal intrapulmonary arteries, real-time RT-PCR and western blotting were done with total RNA and cytosolic protein extracts from rat distal intrapulmonary arteries. Localization of STIM1 and SMαA-positive cells in rat small pulmonary arteries was done by double-immunofluorescence staining to determine the spatial distribution and cellular localization. Compared with the control groups, the expression of STIM1 mRNA and protein increased significantly in the distal intrapulmonary of animals in the 21-day hypoxia treatment group (Figure 2).

### Silencing of STIM1 significantly inhibites PASMCs proliferation under hypoxia

**Figure 3:**
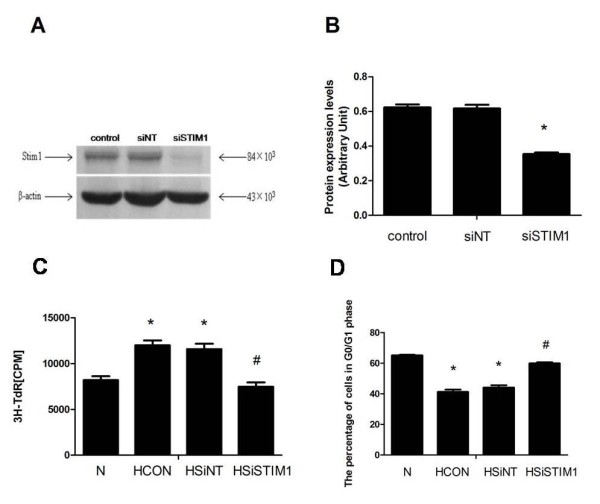
**The effect of STIM1 silencing on rat PASMC proliferation and cell cycle progression under hypoxia.** (**A**). Western blot showing STIM1 and β-actin protein expression from untreated control PASMCs (Control) and PASMCs treated with transfection-nontargeted siRNA (siNT) or siRNA targeted to STIM1 (siSTIM1). (**B**). Mean ratios of STIM1 protein expression relative to that of β-actin, as measured by Western blotting in Control, siNT, and siSTIM1 PASMCs. *: *P* < 0.05 compared to the Control group and the siNT group. The results are expressed as the mean ± SEM of four experiments. (**C**)After transfection with STIM1 siRNA, PASMCs were cultured in hypoxic conditions for 24 hours. [3H]-thymidine (3H-TdR) incorporation was measured to detect PASMC proliferation. (**D**) PASMCs were harvested for flow cytometry-based cell cycle analysis. PASMCs were either left untreated under normoxic conditions (N), left untreated under hypoxic conditions (HCON), treated with nontargeted siRNA under hypoxic conditions (HsiNT), or treated with siRNA targeted to STIM1 under hypoxic conditions (HsiSTIM1). *:*P* < 0.05 compared with the Control group and #:*P* < 0.05 compared with the HCON and HsiNT group. The results are expressed as the mean ± SEM of three experiments.

STIM1 protein expression in PASMCs of siNT group was not significantly different compared with the untreated group. In contrast, siSTIM1 treatment significantly reduced STIM1 protein expression, as compared to siNT treatment or no treatment (P < 0.01) (Figure 3A and B). Next, we investigated the effect of the STIM1 siRNA treatment on PASMCs proliferation under hypoxic conditions. After 24 hours of 3% oxygen treatment, there was a significant increase in PASMCs proliferation, as measured by [3H]-thymidine (3H-TdR) incorporation. Meanwhile, Silencing of STIM1 significantly inhibited hypoxia induced PASMCs proliferation. In addition, there were no significant diffe-rences between siNT-treated group and control group (Figure 3C).

### Silencing of STIM1 significantly reduces the cell cycle progression of PASMCs under hypoxia

As PASMCs proliferation was significantly inhibited by STIM1 siRNA treatment, we investigated whether this inhibition could be due to an alteration in cell cycle progression. Flow cytometry was performed to analyse the phases of the cell cycle for each group. We found that treatment with 3% O_2_ significantly increased PASMCs cell cycle progression, while Silencing of STIM1 significantly inhibited cell cycle progression under hypoxic conditions (Figure 3D).

### Silencing of STIM1 inhibits hypoxia-induced enhancement of SOC/[Ca^2+^]i in PASMCs

**Figure 4:**
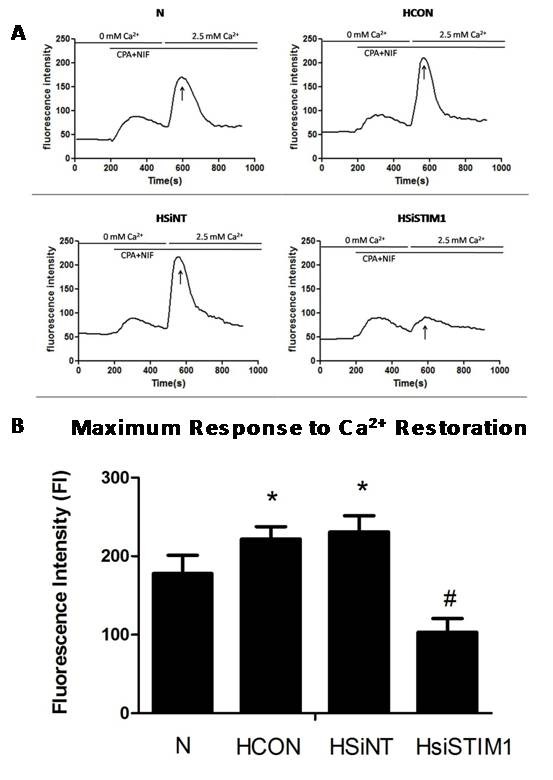
**The effect of STIM1 silencing on hypoxia-induced enhancement of SOC-mediated Ca**^**2+ **^**influx.** After transfection with STIM1 siRNA, PASMCs were cultured in hypoxic conditions for 24 hours. The SOC-mediated PASMC Ca^2+^ influx was measured following stimulation with 10 μM cyclopiazonic acid during the change from Ca^2+^-free conditions to 2.5 mM Ca^2+^. PASMCs were either left untreated under normoxic conditions (N), left untreated under hypoxic conditions (HCON), treated with nontargeted siRNA under hypoxic conditions (HsiNT), or treated with siRNA targeted to STIM1 under hypoxic conditions (HsiSTIM1). **A**: Representative raw trace illustrating the changes in [Ca^2+^]i (presented as fluorescence intensity (FI) in PASMCs in different groups. **B**: Summary of the data illustrated in showing averaged changes in fluorescence after 2.5 mM Ca^2+^ restoration. *:*P* < 0.05 compared with the Control group and #:*P* < 0.05 compared with the HCON and HsiNT group. The results are expressed as the mean ± SEM of three separate experiments.

As hypoxia-induced PASMCs proliferation is typically associated with extracellular Ca^2+^ influx through SOC, we investigated whether the anti-proliferative effects of STIM1 silencing were related to changes in SOC/[Ca^2+^]i as a result of hypoxia. Hypoxia was found to induce a significant increase in peak [Ca^2+^]i levels (P < 0.05) in PASMCs compared to cells from normoxic conditions. The silencing of STIM1 markedly inhibited the hypoxia-induced increase in SOC-mediated Ca^2+^ influx (P < 0.05). These results suggest that STIM1 silencing may exert its anti-proliferative effect by inhibiting the activation of the SOC/[Ca^2+^]i pathway under hypoxic conditions (Figure 4).

### Silencing of STIM1 inhibits hypoxia-induced NFATc3 nuclear translocation

**Figure 5:**
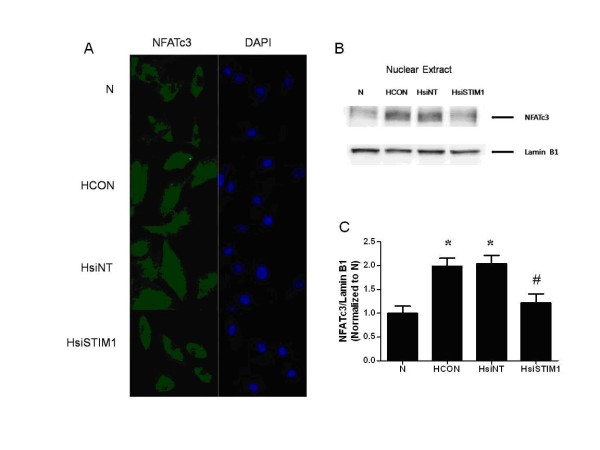
**The effect of STIM1 silencing on hypoxia-induced nuclear translocation of NFATc3 in PASMCs.** NFATc3 staining was visualised by confocal microscopy and immunofluorescence. The primary antibody against NFATc3 was detected using a FITC-conjugated AffiniPure goat anti-rabbit IgG secondary antibody (green). The slides were counterstained with the nuclear dye DAPI (blue). (**A**): Immunofluorescence image of NFATc3 in PASMCs: PASMCs were either left untreated under normoxic conditions (N), left untreated under hypoxic conditions (HCON), treated with nontargeted siRNA under hypoxic conditions (HsiNT), or treated with siRNA targeted to STIM1 under hypoxic conditions (HsiSTIM1). Representative Western blot images (**B**) and summarized data (**C**) for NFATc3 nuclear levels, Lamin B1 was used as a loading control. *:*P* < 0.05 compared with the Control group and #:*P* < 0.05 compared with the HCON and HsiNT group. The results are expressed as the mean ± SEM of three separate experiments.

In normoxic-treated PASMCs, NFATc3 immunostaining in the nucleus was weak which suggested that NFATc3 levels in the nucleus were low and that there was a lack of significant nuclear translocation. Hypoxia treatment significantly stimulated the nuclear translocation of NFATc3, which was indicated by strong NFATc3 staining in the nucleus. Moreover, the silencing of STIM1 significantly attenuated the nuclear translocation of NFATc3 induced by hypoxia (Figure 5A). That silencing of STIM1 inhibits hypoxia-induced NFATc3 nuclear translocation was confirmed by western blot analysis of nuclear extracts(Figure 5B).

## Discussion

Continuous or intermittent hypobaric hypoxia can lead to long-term contraction of the pulmonary artery and structural changes in the pulmonary vascular wall known as hypoxic pulmonary vessel remodelling (HPVR) [20]. HPVR is characterised by thickening of small pulmonary artery wall and muscularizing of pulmonary arteriole, which can result in sustained high pulmonary artery pressure and right ventricular hypertrophy [21]. It has become clear that pulmonary vascular smooth muscle cells (PASMCs) are closely related to the development of pulmonary hypertension, which are regulated by intracellular Ca^2+^ concentrations and calmodulin (CaM) [1]. The intracellular Ca^2+^ concentration has also been suggested to regulate gene expression and cellular proliferation [2,6,22-25]. Intracellular calcium levels in PASMCs are mainly regulated by extracellular calcium influx and the release of intracellular calcium stores. Chelation of extracellular calcium in human PASMCs can significantly inhibit serum or growth-factor induced cell proliferation. The intracellular levels of calcium were significantly higher in proliferating PASMCs than resting PASMCs [26]. These results suggested that a continuous inflow of extracellular calcium is necessary for cell proliferation. The calcium channels found at the plasma membrane, which mediate the influx of extracellular calcium, include voltage-dependent calcium channels (VDCC), receptor-operated calcium channels (ROC), and store-operated calcium channels (SOC). VDCCs are regulated by membrane potential, while SOCs are activated by the depletion of intracellular calcium stores. ROCs can activate G proteins and phospholipase C (PLC), which then hydrolyse phosphatidylinositol 4,5 bisphosphate (PIP2) to generate 1, 4, 5 - trisphosphate (IP3) and diacylglycerol (DAG). IP3 acts on the endoplasmic reticulum (ER) or the sarcoplasmic reticulum (SR) to cause Ca^2+^ to be released from calcium stores and to activate SOC [7,8]. Previous studies have suggested that hypoxia can suppress voltage-dependent potassium channels (Kv) and cause cell membrane depolarisation and the activation of VDCCs. Increased concentrations of intracellular calcium ions can lead to pulmonary vasoconstriction and vascular remodelling [2-5]. However, recent evidence has also indicated that SOCs also play a very important role in the pathogenesis of hypoxic pulmonary hypertension. Vera et al have reported that Ni^2+^ known as SOC blocker can significantly inhibit the proliferation of cultured PASMCs [27,28]. Meanwhile, Lin et al showed that PASMC SOCs are upregulated by chronic hypoxia and contribute to the enhanced vascular tone in hypoxic pulmonary hypertension [6].

The molecular composition and the mechanisms behind the activation of SOCs remained enigmatic for almost 20 years [28,29]. Then, in 2005, using an RNA interference-based high-throughput screen in Drosophila S2 cells with 170 genes, Roos et al. [9] identified the gene for STIM1 as being required for thapsigargin-induced SOC entry. In addition, SOC were not activated in human T cells and HEK293 cells upon the depletion of intracellular calcium stores when STIM1 was silenced. Liou et al. [10] also used an RNA interference-based high-throughput screen in Hela cells with 2,304 genes to identify STIM1 as the key factor necessary to activate SOCs. These experiments indicated that STIM1 was a SOC-specific gene that regulated SOC activity, and the results of these studies indicated that STIM1 may be a novel target for SOC-related diseases. Recently, Lu et al. [14] reported that expression of STIM1 was greater in distal than proximal PASMCs, which may account for the reason why HPV is greater in distal than proximal pulmonary arteries. However, the role of STIM1 in the development of chronic hypoxic pulmonary hypertension especially the relationship between hypoxia induced PASMCs proliferation and STIM1 remains obscure. Therefore, we investigated STIM1 expression level in the distal intrapulmonary vasculature of normal rats and animals exposed to chronic hypoxia. The results demonstrated that a significant upregulation of STIM1 expression was associated with chronic hypoxia-induced PASMC hyperproliferation in the distal intrapulmonary vasculature. Our data indicate that STIM1 protein may be involved in the regulation of hypoxic pulmonary vascular remodelling.

In addition, we used STIM1-specific siRNA to silence the STIM1 expression in rat PASMCs under conditions of hypoxia to investigate the role of STIM1 in the regulation of hypoxia-induced PASMC proliferation. We found that the knockdown of STIM1 expression in PASMCs by RNA interference was highly specific and could be quantitated at the protein level. STIM1 knockdown markedly inhibited SOCE, as measured by the peak [Ca^2+^]i response to the restoration of extracellular Ca^2+^. These findings are consistent with those of other smooth muscles [30,31], including the murine aorta, the human airway, the coronary artery, and the saphenous vein, and confirm that STIM1 contributes significantly to SOCE in pulmonary smooth muscle. Moreover, STIM1 silencing not only inhibited PASMCs proliferation but also reduced the cell cycle progression induced by hypoxia. These results demonstrate that STIM1 is a critical regulator of hypoxia-induced PASMCs proliferation.

Nuclear factor of activated T cells (NFAT) is a Ca^2+^-dependent transcription factor. Elevated levels of intracellular Ca^2+^ increase the activity of the Ca^2+^-calmodulin-dependent phosphatase calcineurin. Activated calcineurin dephosphorylates multiple serine residues within the regulatory region of the NFAT molecule to induce a conformational change in NFAT that exposes nuclear localisation signals and allows for NFAT nuclear import and the subsequent regulation of gene transcription [32]. The NFAT transcription factor family is composed of four well-characterised members including NFATc1 (NFAT2/c), NFATc2 (NFAT1/p), NFATc3 (NFAT4/x), and NFATc4 (NFAT3) [33]. The NFATc3 isoform has specifically been implicated in vasculature development, the maintenance of a contractile phenotype, and the regulation of vascular smooth muscle cell (VSMC) contractility. Recently, Sergio et al. [34] reported that chronic hypoxia induced NFAT transcriptional activity and NFATc3 nuclear translocation in mouse pulmonary arteries. Wang et al. [35] reported that hypoxia-induced NFAT nuclear translocation via the up-regulation of TRPC1 as well as increased SOC-mediated Ca^2+^ influx and the inhibition of the SOC/Ca^2+^/NFAT pathway was involved in the anti-proliferative effect of sildenafil on PASMCs. These results highlight the important role that the SOC/Ca^2+^/NFATc3 pathway plays during the development of hypoxic pulmonary hypertension. In this study, we hypothesised that STIM1 knockdown would inhibit the proliferation of PASMCs under hypoxia via the inhibition of the SOC/Ca^2+/^NFATc3 pathway. By observing the effect of STIM1 knockdown on PASMC SOC-mediated Ca^2+^ influx and NFATc3 nuclear translocation under hypoxic conditions, we found that hypoxia could increase SOC-mediated Ca^2+^ influx and promote NFATc3 nuclear translocation. Thus, STIM1 silencing could significantly inhibit SOC-mediated Ca^2+^ influx and NFATc3 nuclear translocation. These results indicate that STIM1 is an essential regulator of the SOC/Ca^2+^/NFAT pathway, which plays an important role in hypoxia-induced PASMC proliferation.

In summary, this report presents in vivo and in vitro evidence to indicate that STIM1 may be involved in the development of hypoxic pulmonary vessel remodelling. As hypoxia induces PASMCs proliferation through a STIM1-dependent mechanism and the SOC/Ca^2+^/NFAT pathway, STIM1 may represent a novel therapeutic target for the prevention of hypoxic pulmonary hypertension.

## Competing interests

The authors declare that they have no competing interests.

## Authors’ contributions

XH contributed in all the data experiments, analysis and elaborated the figures. JC performed the Western blot, their analysis and contributed to immunofluorescence experiments. YL contributed in qRT-PCR analysis and amelioration of the manuscript. FL and GX contributed in analyzed in vivo measurements and cell culture experiments. YG designed the study, supervised the overall study and wrote the manuscript. All authors have read and approved the manuscript.
